# Extracellular Vesicles and Their Associated miRNAs as Potential Biomarkers in Intracranial Aneurysm

**DOI:** 10.3389/fmolb.2022.785314

**Published:** 2022-06-20

**Authors:** Yuman Li, Jiahao Wen, Dingyue Liang, Haitao Sun

**Affiliations:** ^1^ Clinical Biobank Center, Microbiome Medicine Center, Department of Laboratory Medicine, Zhujiang Hospital and the Second Clinical Medical College, Southern Medical University, Guangzhou, China; ^2^ Neurosurgery Center, The National Key Clinical Specialty, The Engineering Technology Research Center of Education Ministry of China on Diagnosis and Treatment of Cerebrovascular Disease, Guangdong Provincial Key Laboratory on Brain Function Repair and Regeneration, The Neurosurgery Institute of Guangdong Province, Zhujiang Hospital, Southern Medical University, Guangzhou, China; ^3^ Key Laboratory of Mental Health of the Ministry of Education, Guangdong–Hong Kong–Macao Greater Bay Area Center for Brain Science and Brain–Inspired Intelligence, Southern Medical University, Guangzhou, China

**Keywords:** intracranial aneurysm (IA), exosomes, miRNA—microRNA, blood, biomaker

## Abstract

Intracranial aneurysms (IA) are abnormal expansions of the intracranial arteries. Once it ruptures, the mortality and disability rate are high. The cost of imaging examinations is high, and rupture risk cannot be predicted, making it difficult for high-risk groups to be screened and prevented. Thus, clinically effective biomarkers are required to screen high-risk groups, estimate the risk of rupture, and determine the appropriate early intervention step. This article introduces the current research and application of exosome-derived microRNA (miRNA) as biomarkers of intracranial aneurysms and their limitations, which can give researchers a general overview of the research in this field. It can also serve as a reference point for selecting related research directions.

## Introduction

Intracranial aneurysms (IA) are caused by the reduction of localized structural integrity in the arterial wall, because of damage to the internal elastic membrane and disruption of the media. A population without comorbidities is estimated to have a prevalence of 3.2% (95% confidence interval 19–52) with an average age of 50, and males occupy 50% of the population (Bederson et al., 2009). IA ruptures occur approximately 9.1 times per 100,000 annually, and mortality rates are close to 40%. Nonetheless, 46% of survivors will also have disabilities or long-term cognitive impairment due to multiple complications (Heo et al., 2020; Cao et al., 2021; Ma et al., 2021).

In most cases, early IAs are asymptomatic, and vascular imaging is a common early screening and diagnostic tool. The Digital Silhouette Angiography (DSA) is the gold standard for diagnosing IA (1), but it is intrusive and time consuming. The use of non-invasive techniques like computed tomography angiography (CTA) in IA diagnostics is growing, but CTA is less sensitive to smaller aneurysms and requires a large amount of radiation and contrast agents, both of which can adversely affect patients with impaired kidney function or those who are sensitive or allergic to contrast agents (Pradilla et al., 2013). Magnetic resonance angiography (MRA), another non-invasive technique, has no such disadvantage, and it is as accurate as CTA in diagnosing intracranial aneurysms, but it is less specific (Sailer et al., 2014). When assessing the stability of aneurysms by imaging, though the risk of rupture increases with aneurysm size, the study found that most ruptured aneurysms had a small diameter, which could be explained by the fact that they developed faster than expected and ruptured earlier in the expansion period (You et al., 2010). As a result, the current widely used methods of early screening and evaluation have limitations in sensitivity, specificity, and cost-effectiveness, indicating that finding sensitive, highly specific, minimally traumatic, low-cost methods to detect early IAs and assess the risk of rupture has become a new trend and hot topic.

The exosome is a type of extracellular vesicle released by the plasma membrane (PM) of various cells into various body fluids, including plasma, breast milk, saliva, sweat, tears, semen, and urine (Černá et al., 2019; Gareev et al., 2020). The lipid bilayer membrane of exosomes protects RNA, microRNA (miRNA), proteins, and other signal molecules from degradation (Liu et al., 2019). MiRNAs are epigenetic regulators among these molecules. Different biological processes such as angiogenesis, growth, and differentiation can be affected by these molecules. Accordingly, miRNA types and content may be altered in the early stages of various diseases (Liu et al., 2019). Studies have shown that the levels of certain plasma exosome-derived miRNA molecules vary between normal individuals and individuals with unruptured intracranial aneurysms, between patients with ruptured and unruptured intracranial aneurysms, and between severe and mild patients (Liao et al., 2020). In this way, plasma secreted miRNA may play a role in the formation and rupture of IA, and finally express itself in changes in its expression content. By detecting changes in plasma exosomal miRNA content, a non-invasive, convenient way to make an early diagnosis and predict the risk of rupture is possible in intracranial aneurysm occurrence and development.

In addition to imaging, IA patients have no clear non-invasive biomarkers (Ma et al., 2021). Hence, the understanding of clinically relevant diagnostic markers may contribute to the development of better diagnostic methods to identify IAs and their tendency to rupture. Numerous studies have shown that exosomes may serve as a biomarker for detecting intracranial aneurysms early, predicting rupture, and assessing the severity of the disease. In this review, we will discuss the use of exosomes in intracranial aneurysms.

## MiRNA in Blood Derived Exosomes Exhibits Favorable Clinical Biomarker Properties

In recent years, many studies have been conducted on biomarkers of intracranial aneurysms, including protein, miRNA, long non-coding RNA (lncRNA), circular RNA (circRNA), and so forth. Compared to nucleic acid biomarkers, protein biomarkers have poor stability and difficulty amplification. Among nucleic acid biomarkers, miRNA is far more studied than lncRNA and circRNA.

MiRNA is a type of highly conserved, non-coding small RNA that ranges in length from 18 to 24 nucleotides (Ma et al., 2021). MiRNA regulates gene expression at the post-transcriptional level by interacting with the 3′-untranslated regions (3′UTRs) of target mRNAs (Cao et al., 2021). MiRNA is a regulator of essential biological processes in animals, which affects gene expression by recognizing homologous sequences and interfering with transcription, translation, or epigenetic processes. Multisteps are involved in the generation of mature miRNAs in animals. MiRNA genes are usually transcribed into primary miRNA (pri-miRNA) by RNA polymerase II, and then cleaved into hairpin precursor miRNA (pre-miRNA) by nuclear RNase III Drosha, and then exported to the cytosol by exportin-5 (9). Pre-miRNA is cut into double stranded miRNA in the cytoplasm by Dicer, and one arm is loaded into Argonaute (AGO) protein in RNA-induced silencing complex (RISC), which serves as a guiding sequence for binding to mRNAs (Liu et al., 2019). MiRNA and its target mRNA are recognized by partial base complementary pairing. The first eight nucleotides at the end of miRNA 50, called seed region, control this process. (Vlak et al., 2011). Seed sequence is the main factor determining the specificity of target gene, and the relatively short seed region makes miRNA have multiple target mRNAs (Sun et al., 2020). The ability of miRNAs to interact with multiple target genes allows them to influence a wide range of biological processes and pathways (Liao et al., 2020).

Up to two-thirds of human protein-coding genes are regulated by MiRNA, which also participates in a wide range of biological processes, including embryonic development, cell differentiation, apoptosis, metabolism, and tumorigenesis (Friedman et al., 2008). Many experimental studies have shown that miRNA plays an key role in vascular diseases by regulating the functions of vascular smooth muscle cells and endothelial cells, such as proliferation, migration, apoptosis, angiogenesis and extracellular matrix (ECM) protein synthesis and secretion (Kloosterman and Plasterk, 2006; Cheng et al., 2009; Dentelli et al., 2010; Maegdefessel et al., 2012; Rutnam et al., 2013). It is without a doubt that tissue-specific and phase-specific expression of miRNAs play an incredibly significant role in the cell cycle. According to the different expression levels of miRNA in different tissues, its physiological functions may differ depending on the tissue. When internal and external factors act on the arterial wall, the original balance is lost. Changes in the structure of the arterial wall and the formation or rupture of aneurysms can affect miRNA expression levels and consequently affect the response of tissues and cells to stimulation. Using microarray hybridization, Hengwei Jin and colleagues observed differences in serum miRNA expression levels between normal and aneurysm patients and between different stages of aneurysm, demonstrating the phase-specific nature of miRNAs and displaying aneurysm growth. This suggests that serum miRNAs may not only affect the development and occurrence of intracranial aneurysms, but may also be associated with the rupture of intracranial aneurysms as well (Pritchard et al., 2012). Then again, miRNAs exist stably in a series of specimens, including serum, plasma, urine, and formalin-fixed tissues, and their physicochemical properties are similar in fresh and frozen serum and plasma (Pritchard et al., 2012). Therefore, miRNA is expected to be a new clinical biomarker.

MiRNA is expressed in the cell, but it can be secreted out of the cell to become circulating miRNA in three ways: 1) Passive secretion by damaged cells due to apoptosis or necrosis; 2) Active secretion by extracellular vesicles (EV); 3) Active secretion by RNA-binding protein-dependent pathways (Zaporozhchenko et al., 2018).

The exosome is a type of extracellular vesicle. The minimum diameter of exosomes is typically about 30–100 nm, but when stimulated by miscellaneous physical or chemical factors, the plasma membrane buds inward in a ceramide-triggered budding process, producing early endosomes (EEs). In the late stage, the further inward budding of the endosomal membrane induces multivesicular bodies (MVBs). MVBs accumulate intracellular intraluminal vesicles (ILVs), which show dynamic changes in response to pathology or physiological conditions. After fusion with the plasma membrane, MVBs can enter lysosomes, where they are degraded by proteasomes. In addition, they can secrete ILVs containing transmembrane proteins and other functional cytosolic components such as miRNA and mRNA into extracellular space. The released ILVs is called “exosomes” (Liao et al., 2020). Exosomes are secreted by most types of cells in the body and are found in various body fluids including plasma, cerebrospinal fluid, saliva, breast milk and urine. Their molecular composition reflects the physiological or pathological changes occurring in their cells or tissues of origin. Exosomes can bind to recipient cells and release their contents into them. They also play an important role in cell-to-cell communication by transferring proteins, RNAs, and lipids from one cell to another (Bederson et al., 2009).

Several studies have demonstrated that most circulating miRNAs come from apoptotic and necrotic cells (Cortez et al., 2011; Pritchard et al., 2012). If miRNAs in circulating blood are observed directly, the results are easily influenced by miRNAs from other sources. This makes it difficult to accurately reflect the pathophysiological process of vascular diseases. Research on miRNA in circulating blood as a biomarker for IA diagnosis has been inconsistent (Jin et al., 2013; Li et al., 2014; Meeuwsen et al., 2017). The sources of heterogeneity in miRNAs, such as race, gender, lifestyle, and inflammatory status (Wagner et al., 2016; Pheiffer et al., 2018; Ludwig et al., 2019; Zhao et al., 2019),as well as the different types of samples (Nakajima et al., 2000) all have the potential to influence the research results. However, the most significant factor is the source heterogeneity in miRNAs. Furthermore, determining the origin of specific miRNAs in circulation under pathological or physiological conditions is a major challenge (Endzeliņš et al., 2017; Murray et al., 2017), which further compromises the feasibility of direct detection of circulating blood miRNAs.

Instead, miRNA is released by extracellular vesicles such as exosomes or protein-miRNA complexes like Argonaute protein and lipoprotein, and miRNA circulating in the body can have important biological effects (Gareev et al., 2020). Because of the protection provided by the lipid bilayers of exosomes, miRNAs packaged within exosomes retain their stability and evade degradation by nucleases (Bei et al., 2017). (Endzeliņš et al., 2017; Murray et al., 2017) Exosomes also seem to be more sensitive than free miRNAs and cellular miRNAs. A study found that miR-126 levels in circulating exosomes decreased significantly 3 h after transient and permanent ischemia, but that levels of free miR-126 decreased only after permanent ischemia and not after transient ischemia (Xia et al., 2019). As a result, exosome-derived miRNAs are considered to be better biomarkers.

Exosomes derived from blood seem to be the most reliable source of exosomes. It is easy to collect exosomes from urine and saliva, and they contain a high level of exosomes from different organs and tissues. However, their exosome concentration and overall composition are largely determined by fluid and food intake, while blood and cerebrospinal fluid exosomes are generally stable. Though cerebrospinal fluid produced in the cerebral choroid plexus theoretically has the most relevant “content” to the brain, there are several problems: 1) the collection method is invasive; 2) sample sizes are small, limiting the amount of exosomes that can be extracted; 3) if the aneurysm ruptures, the blood may pollute the cerebrospinal fluid. The method of obtaining circulating blood also has the advantages of being convenient, non-invasive, and low-cost, making it suitable for population screening. Blood derived exosomes contain only part of the information about intracranial aneurysms, and the separation of that information will be crucial in the future.

## Study of Circulating Exosome-Derived miRNA in Intracranial Aneurysms

In this review, we divided the research on secreted miRNA in intracranial aneurysms into development and prediction (early diagnosis, rupture). Experimental studies are mostly involved in the former, while screening different secreted miRNAs in the population is more of a concern in the latter.

### The Role of Circulating Exosome-Derived miRNA in the Development of Intracranial Aneurysms

It has been found that the onset and development of IA is a complex pathophysiologic process, influenced by racial factors, smoking habits, sex, and hemodynamics, as well as other congenital and acquired factors; however, the mechanism for its occurrence remains unknown. Research studies have shown that inflammation has an effect on the formation and development of intracranial aneurysms. Endothootin malfunction is considered the first step in the formation of IAs, and can be caused by smoking, hypertension, local hematological disorders, and genetic factors. Oxidative stress is a major cause of endothelial damage, and free radical accumulation occurs because of increased production and/or decreased removal of free radicals (Starke et al., 2013). The next coordinated inflammatory response involves macrophages, fat cells, T cells, and many cytokines and inflammatory media. This inflammatory response leads to phenotype regulation of vascular smooth muscle cells, which are the main matrix synthesis cells in arterial wall media (Nakajima et al., 2000). As a consequence, vascular smooth muscle cells destroy the internal elastic layer, causing collagen synthesis to become imbalanced and the extracellular matrix to remodel (Ali et al., 2013).

Currently, few experiments have been performed on secreted miRNA in intracranial aneurysms. Feng, Z., et al. studied the role of exosomes derived from tumor-related macrophages (TAM) that contain miR-155–5p in IA formation. Their study found high miR-155–5p expression in TAM-derived exosomes. Furthermore, they found that miR-155–5p contributes to the proliferation and migration of blood vessel smooth muscle cells. Exosomes derived from TAMs transfected with miR-155–5p inhibitors inhibit the proliferation and migration of smooth muscle cells *in vitro*. According to a previous study (Sarzani et al., 2020), exosome-mediated miR-155 produced by vascular smooth muscle cells spread from vascular smooth muscle cells to endothelial cells, thereby compromising the integrity of the endothelial barrier, resulting in increased endothelial permeability and atherosclerosis progression.

Yang et al. (2021) first co cultured vascular endothelial cells (VECs) with ectoplasts derived from bone marrow mesenchymal stem cells (MSCs) and transfected mir-144–5p simulant or inhibitor. When mir-144–5p analogue was injected into IA rats, the rats showed greater numbers of endothelial cells and fibroblasts, less smooth muscle cells but better arranged, larger lumen expansions, less smooth muscle layers, and thinner vascular walls. The condition of rats injected with mir-144–5p inhibitor deteriorated further. Additional experiments showed that the effect of mir-144–5p on endothelial cells was mediated by phosphatase and tensin homologue (PTEN). The mir-144–5p analog increased cell viability, while overexpression of PTEN weakened the effect, thus promoting apoptosis and clogging the vascular endothelial cells.

Sun et al. (2020) used ultrahigh-speed centrifugation to extract exosomes from human bone marrow-filled stem cells. Results showed that the exosome of bone marrow interstitial stem cell source improved pathological remodeling of the IA wall, increased the contraction phenype of smooth muscle cells, inhibited the secretory phenotype of smooth muscle cells, decreased the number of Th17 cells, and maintained the balance of Th17/Treg. Further research has demonstrated that the outer hsa-miR-23b-3p of the source of the bone marrow interstitial charge stem cell can maintain the Th17/Treg balance by suppressing THEI3k/Akt/NF-B signal pathway targeting KLF5, thereby inhibiting the formation of IA.

Although these experiments illustrate a possible mechanism of exosomal miRNAs, the way these miRNAs interact under more complex *in vivo* conditions remains a mystery. They should be confirmed by more rigorous experiments in the future. Additionally, these results may help predict biomarkers. Therefore, differential miRNA expression in the population can provide evidence to support these experiments.

### Application of Circulating Exosome-Derived miRNA in Early Diagnosis of Intracranial Aneurysms

In a study comparing 84 IA patients to 45 healthy individuals, Yang et al. (2021) found a significant decrease in exosome miR-144–5p levels in serum in ruptured intracranial aneurysm (RA) and unruptured intracranial aneurysm (UA) patients. To identify the plasma exosomes extracted from the blood sample, QinYansheng (Qin, 2019) used a commercial exosome extraction kit to extract the exosomes from the blood sample, and observed the morphology of the extract using a transmission electron microscope. In view of the fact that the isolated vesicular substances accord with the typical morphological characteristics of exosomes, it may be assumed that the vesicles from plasma samples are exosomes. By using high-throughput sequencing, he obtained the plasma exosomal miRNA expression profile in 12 patients with intracranial aneurysms (4 unruptured, 8 ruptured (4 mild, 4 severe))and 4 healthy individuals, and he then sequenced these miRNAs in the control group composed of 20 healthy individuals as well as in the experimental group of aneurysmal subarachnoid hemorrhage (aSAH) made up of 39 individuals (divided into mild). Differentially expressed exosomal mir-202–5p was screened. Significant differences were found in the expression of miR-202–5p in the normal group and in patients with unbroken intracranial aneurysms (decrease), unbroken and ruptured groups (up), severe and mild (up). However, there was no significant difference in expression between normal and fractured groups. Due to the possibility of experimental errors caused by a small sample size, the author conducted an extended sample polymerase chain reaction (PCR) verification of miR-202–5p; the results showed that the expression of miR-202–5p differed among groups. The author performed a further biological analysis of miR-202–5p, and GO enrichment analysis revealed that miR-202–5p was involved in mainly protein phosphorylation, positive and negative regulation of transcription and translation, gene expression regulation, maintenance of myelin in the central nervous system, and myosin cytospinal tissue. There are three main cell components - nucleotysis, endometrium, and Gorky membrane. The main molecular function is uterine protein ligase activity, sequence-specific DNA binding, and mRNA binding. KEGG’s enrichment pathways (hepatitis B, prostate cancer, colorectal cancer, HTLV-I infection, insulin resistance) suggest that miR-202–5p is involved in the pathological process of a variety of diseases. This is consistent with recent findings that miR-202 promotes or inhibits tumor growth through cell proliferation, apoptosis, etc. However, the article failed to discuss the abundance and structural integrity of the extracted exosomes, and the purity of the exosomes extracted by this method remains unknown.

Similarly, Liao et al. (2020) took a blood sample from an elbow vein, using an exosome extraction commercial kit to extract plasma exosomes in the sample, and identified the extract by transmission electron microscope and protein imprint analysis. Observe that the shape and size of the isolated vesicles correspond to that of previously reported exosomes, and that the vesicles express exosome specific proteins CD9, Hsc70, and TSG101. It can thus be concluded that the extracted vesicles are exosomes with complete structure, uniform particle size, and high abundance, which can be used for further sequencing of miRNAs. As a result of the initial screening phase, he identified 181 differentially expressed exosome miRNAs. Nevertheless, there were no significant differences in Wayne diagrams for groups healthy control (HC), UA, and RA. Subsequently, in the validation part of the experiment, the researchers randomly selected some of the above differences in miRNA for quantitative real time polymerase chain reaction (qRT-PCR) validation in larger samples. There was a significant increase in miR-145–5p in UA and RA compared to HC, a significant increase in miR-145–5p expression in RA compared to HC and UA, and a significant increase in miR-29a-3p in UA and RA (*p* < 0. 001). Since the sample size is too small and the genetic criteria defined differ, the authors prefer qRT-PCR results. Based on the ROC curve, it was found that miR-29a-3p had an AUC of 0.791 (95% CI, 0.702–0.879) in differentiating between aneurysms, and an AUC value of 0.737 (95% CI, 0.617–0.85), and multifactoral logistic regression analysis indicated that miR-29a-3p and miR-145–5p are independent diagnostic markers (*p* < 0.05). Unusually, Xu Gangzhu et al. (Xu et al., 2021) confirmed significant differences in miR-145–5p in the health group as well as the IA group.

A summary of how circulating exosome-derived miRNA is used in the early diagnosis of intracranial aneurysms is provided in Table 1.

**TABLE 1:** Summary of circulating exosome-derived miRNA in early diagnosis of IA.

biomarker	Author and year	Sample source	Number of patients (diagnosis)	Mean age (SD)/(Range)	miRNA in plasma derived exosomes	UA	RA
miR-145–5p	Xu Gang-Zhu, 2021	Blood samples from the elbow vein	Number = 110 (32RA,78UA); control = 92(HC)	patients:59.62 ± 8.63; control = 57.02 ± 9.94	miR-145–5p was significantly increased in RA and UA compared with the HC	↑	↑↑
miR-145–5p	Liao B, 2020	Blood samples from the elbow vein	Number = 12 (39RA,30UA); control = 30(HC)	UA: 54.9 ± 19.1; RA: 56 ± 14.5; control = 42 ± 13.1	miR-145–5p was significantly increased in RA and UA compared with the HC		
miR-29a-3p	Liao B, 2020	Blood samples from the elbow vein	Number = 12 (39RA,30UA); control = 30(HC)	UA: 54.9 ± 19.1; RA: 56 ± 14.5; control = 42 ± 13.1	miR-29a-3p was significantly increased in RA and UA compared with the HC	↑	↑
miR-202–5p	Qin Yan-Sheng, 2019	Blood samples from the elbow vein	Number = 39 (20 Severe group, 19 mild group); control = 20(HC)	Not mentioned	miR-202–5p was significantly reduced in RA and significantly increased in UA compared with the HC	↓	↑

### Application of Circulating Exosome-Derived miRNA in Predicting Rupture of Intracranial Aneurysms

In Liao Bao (Liao et al., 2020) ‘s exploratory study, the next-generation sequencing (NGS) method was used to determine miRNA expression profile characteristics of circulating exosomes in IA patients, and the sequencing results were confirmed by qRT-PCR. Among the validated miRNAs, miR-145–5p is up-regulated in RA compared to HC and UA. The analysis revealed that the AUC value of miR-145–5p as a diagnostic marker for aSAH or predictor of aneurysm rupture was 0.737 (95% CI, 0.617–0.856), and the expression levels of miR-145–5p expression were not related to age, gender, hypertension, diabetes, smoking, drinking and location of aneurysms (*p* > 0.05), but were related to the size of aneurysm (*p* < 0.05). An increase in miR-145 expression instructs vascular smooth muscle cells to differentiate and, subsequently, alters the formation of neointima, suggesting that miR-145 is an important phenotypic regulator of vascular smooth muscle cells and can distinguish between unruptured and ruptured aneurysms, which may be an ideal marker for predicting rupture risk.

Also, in an exploratory study conducted by Xu Gang Zhu and his colleagues (Xu et al., 2021), plasma-derived miRNA from IA patients was also sequenced and analyzed. Firstly, they take a blood sample from the elbow vein, then separate the plasma and isolate the exosomes. The exosomes were identified by transmission electron microscopy. Subsequently, the plasma exosomal miRNAs were detected by real-time quantitative reverse transcription polymerase chain reaction. In the IA group, miR-145–5p expression levels were significantly higher than in the healthy group (5.32 ± 1.19), (*p* < 0.05). The expression of plasma exosome-derived miR-145–5p in the ruptured aneurysm group was higher (13.92 ± 0.31), when compared to the unruptured aneurysm group (10.69 ± 1.27). The AUC value for miR-145–5p in the assessment of aneurysm rupture in IA patients was 0.765, the best boundary value was 10.145, the sensitivity was 80.80%, and the specificity was 81.20%. Another risk factor for rupture of aneurysms in IA patients was over 60 years of age, diabetes, hyperlipidemia, aneurysm diameter wider than 5 cm, and miR-145–5p expression in plasma exosomes greater than 11.63. The study demonstrated that miR-145–5p overexpression led to imbalances between vascular smooth muscle cell (VSMC) proliferation and apoptosis, resulting in IA. The test has certain value in assessing aneurysm rupture risk, and it is expected to become the ideal marker for predicting the risk of IA rupture. Compared to Liao Bao’s experiment, however, this method of extracting and identifying exosomes cannot explain whether their abundance and structural integrity can be good enough to sequence miRNAs.

Qin Yan-Sheng (Qin, 2019) analyzed the miRNA expression profile of plasma-derived exosomes from 12 patients with IA (4 cases of unruptured aneurysm, 4 cases of mild disease, and 4 cases of severe disease) and 4 healthy subjects through high-throughput sequencing, and identified miR-202–5p with significantly different expression and validated it by qRT-PCR. Exosome-derived miR-202–5p expression was up-regulated in ruptured aneurysms as compared to unruptured aneurysms (*p* < 0.05). The relative expression level of miR-202–5p was higher in the severe group than in the mild group (*p* < 0.05). There has been no research on miR-202 in patients with IA, however, the research suggests that the plasma exosome-derived miR-202–5p is highly expressed in patients with aSAH (ruptured aneurysm), which may become an evaluation index of the severity of aSAH patients and an ideal marker for predicting rupture risk. In the above description of this experiment, it can be seen that the biological process, cell components, and molecular functions targeted by mir-202–5p suggest that mir-202–5p could be a part of the rupture process of intracranial aneurysms. The enrichment pathway of KEGG indicates that mir-202–5p is involved in the physiological process of many diseases.

The application of circulating exosome-derived miRNAs in predicting intracranial aneurysm rupture is summarized in Table 2.

**TABLE 2:** Summary of circulating exosome-derived miRNA in predicting rupture of IA.

biomarker	Author and year	Sample source	Number of patients (diagnosis)	Mean age (SD)/(Range)	miRNA in plasma derived exosomes	UA	RA
miR-145–5p	Liao B, 2020	Blood samples from the elbow vein	Number = 12 (39RA,30UA); control = 30(HC)	UA: 54.9 ± 19.1; RA: 56 ± 14.5; control = 42 ± 13.1	miR-145–5p was significantly increased in RA compared with the UA and HC	↑	↑↑
miR-145–5p	Xu Gang-Zhu, 2021	Blood samples from the elbow vein	Number = 110 (32RA,78UA); control = 92(HC)	patients:59.62 ± 8.63; control = 57.02 ± 9.94	miR-145–5p was significantly increased in RA compared with the UA and HC		
miR-202–5p	Qin Yan-Sheng, 2019	Blood samples from the elbow vein	Number = 39 (20 Severe group, 19 mild group); control = 20(HC)	Not mentioned	miR-202–5p was significantly increased in RA compared with the UA; miR-202–5p was increased in the severe group compared to the mild group	↓	↑

## Discussion

Biomarkers provide critical information on the diagnosis and treatment of disease. An ideal biomarker should have the following properties (Liao et al., 2020) 1) Easy specimen collection, small harm to subjects, easy to operate, 2) stable chemical properties, useful for *in vivo* and external use, 3) high sensitivity, reliable for early diagnosis of diseases, 4) content changes and disease development, and effects at all levels are causal, 5) high specificity and early warning. In the preceding article, we discussed the characteristics of circulatory exosome miRNA as a biomarker.

Despite the fact that miRNA is still in its infancy as a biomarker, there are currently products in clinical use and others in preclinical and clinical trials. For example, circulating platelet miRNAs are novel biomarkers for intrinsic and on-treatment platelet reactivity. Exosomes are released during platelet activation and can be found in plasma and serum. Case studies can provide valuable information on the path from discovery to clinical application of miRNA biomarkers (Willeit et al., 2013). Similarly, the current studies of circulatory exosome miRNAs as biomarkers in intracranial aneurysms are in their infancy, but other vascular diseases (atherosclerosis, etc) can provide valuable experience as biomarkers in the clinical setting.

The large-scale application of circulatory exosome miRNA as a biomarker in clinical practice still faces some challenges.

First, it is the source of exosomes. In the review mentioned above, the heterogeneity of miRNA sources was suggested as a significant interfering factor. This led to inconsistent results of previous studies on miRNA in circulating blood as an IA diagnostic biomarker. As a matter of fact, exosomal miRNAs also face the same problem. However, compared to the disorder of circulating miRNAs, the functionality of exosomal miRNAs seems more likely to reveal their origin. The composition of exosomes is not random but determined by the accumulation of specific cell markers (Mathivanan et al., 2010). Therefore, exosomes can be preliminarily identified by these different specific cell markers. A recent technology called the Proximity Barcoding Assay (PBA) that analyzes the heterogeneity of exosomes is expected to help resolve the source problem. In this technique, researchers encode individual exosomes by using micrometer-sized single-stranded DNA clusters containing hundreds of copies of unique DNA motifs generated by rolling circle amplification (RCA). The protein components on the surface of a single exosome are converted into DNA sequence information by binding antibody DNA conjugates. The binding antibody DNA conjugates contain random tag sequences repeated in each RCA pipeline. Following PCR amplification, there is next-generation sequencing that determines the combined position of the surface protein of one exosome, based on the identity information gathered from the DNA chains (Wu et al., 2019).

The second thing is that in the literature we retrieved, whether it is differential comparisons of particular miRNA types or matching only known miRNAs following high-throughput sequencing techniques, we still aren't certain about unknown miRNA expressions, which need to be clarified in future studies.

Third, large numbers of biomolecules and protein aggregates in the body fluid may interfere with the detection and analysis of exosomes. Therefore, the main problem in clinical application of exosomes is the inability to isolate purified exosomes from the homogeneity of other subtypes of exocellular vesicles (microbubbles and other soluble contaminants) (Liao et al., 2020). Currently, methods based on exosome density, size, and surface protein, such as supercentrifugation, ultrafiltration, precipitation, chromatography, immunomagnetic beads, or kit extraction, are available (Théry et al., 2002; Li et al., 2017). Nonetheless, these techniques have some disadvantages, and the exosome separation and purification technologies still need to be improved.

Also, before using exosomal miRNA as a clinical biomarker for large-scale disease screening, it is imperative to understand the sensitivity and specificity of the screening technology. The present method of detecting miRNA differences in intracranial aneurysm exosomes relies on high-throughput sequencing technology combined with qRT-PCR. With high-throughput sequencing technology, also known as “next-generation” sequencing technology, its principle is to randomly fragment the DNA (or cDNA) to be detected, build a library, perform extension reactions on thousands of clones within the library, detect the fluorescence signal, and then obtain the sequence information. It has become possible to study miRNA expression profiles comprehensively and effectively thanks to the rapid development of this technology. It has gradually become a preferred method in the medical field due to its small sample size, short time-consuming, low cost, and the ability to accurately identify thousands of genes at once (Liao et al., 2020). Research by Heming Wu et al. found that miR-150–5p, miR-576–3p, and miR-4665–5p could distinguish recurrence of breast cancer patients from breast cancer patients who had not recurred, using high-throughput sequencing technology (Wu et al., 2020). Moreover, NGS overcomes the limitations of gene chips, as it can be used to discover new miRNAs and to confirm existing ones, as well as evaluate expression differences between samples (Stevanato et al., 2016). Juan Liao et al. studied the differences in miRNA expression profiles between human esophageal cancer cells and exosomes, determined by Solexa high-throughput sequencing. The study found that 342 and 48 known miRNAs were identified in cells and exosomes, and 64 and 32 new miRNAs were predicted. The miRNA profiles of several exosomes were similar to their parent cells, which not only confirmed and explained the source of exosomes, but also provided new insights into the characteristics of miRNA expression profiles in esophageal cancer-derived exosomes (Liao et al., 2014). It is particularly critical to select an appropriate target gene screening method before applying high-throughput sequencing for subsequent analysis of data (Liao et al., 2020). Essentially, high-throughput sequencing based on miRNA expression analysis and array-based comparative genomic hybridization will make it easier to study differences in miRNA expression profiles in exosomes from different sources and guide researchers to develop better clinical auxiliary diagnostic tools.

Additionally, if exosomal miRNAs are used in disease screening on a wide scale, whether there are differences within and between individuals in the population cannot be ignored. Research on exosomal miRNA is currently based on the mechanisms and differential genes of specific diseases. However, differences in the expression of exosomal miRNA within and between individuals are rarely studied. Perhaps we can get some inspiration from the study of the inter-individual variability of circulating miRNAs.

First, the review mentioned earlier that “race, lifestyle, gender, inflammation (Wagner et al., 2016; Pheiffer et al., 2018; Ludwig et al., 2019; Zhao et al., 2019) and even different sample types (Nakajima et al., 2000) ” will affect the content of circulating miRNAs. The study of Rekker, K et al. found that the expression level of circulating miRNAs in healthy women did not change significantly during the menstrual cycle; however, the number of miRNAs detected varied significantly between individuals; Figueredo Dde et al. (de Siqueira Figueredo et al., 2015) also found that miR-16 and miR-181a expression in subjects varied by individual. In a recent study (Wan et al., 2015) it was found that 16 miRNAs in the cerebrospinal fluid of patients with major depression were significantly altered when compared with healthy controls. It is noteworthy that hyejin Yoon et al. (2017) presented strong evidence for the first time by demonstrating the immutability of the basic level of most miRNAs in cerebrospinal fluid under normal physiological conditions, suggesting that miRNAs are reliable candidate biomarkers for neurological diseases. In addition, the authors found some highly variable inter- and intra-individual miRNAs. For the significant reduction of mir-451a in one participant, the author measured four others (mir-30a-5p, mir-33a-5p, mir-139–5p, and mir-451a) among the 16 miRNA biomarkers reported by Wan et al. (2015). And further examined the levels of these miRNAs in the participant. Similarly, the four miRNAs observed by Wan et al. (2015)), showed up-regulated or down-regulated expression levels. These findings suggest that the emotional state of the participant could affect miRNA expression in cerebrospinal fluid. These findings support the idea that miRNAs circulating in the blood differ from individual to individual. It is still unknown, however, whether functional exosomes also have these differences. These results should be further confirmed in circulating exosomes in the future.

## Conclusion

MicroRNAs in exosomes play a crucial role in the development of intracranial aneurysms. Therefore, exosome micronucleic acid is a promising biomarker for predicting ruptured intracranial aneurysms. In this paper, we summarize the development of exosomes in intracranial aneurysms and demonstrate their potential use in the early diagnosis of the disease and prediction of rupture risk (Tables 1, 2). Exosomes, however, do not fully understand how intracranial aneurysms in the brain form, which hinders their application in diagnosing and treating intracranial aneurysms. In the future, an understanding of the exosomes in intracranial aneurysms will provide a basis for diagnosis and treatment of intracranial aneurysms, potentially bringing about breakthroughs.
